# Five Years of GenoTyphi: Updates to the Global *Salmonella* Typhi Genotyping Framework

**DOI:** 10.1093/infdis/jiab414

**Published:** 2021-08-28

**Authors:** Zoe A Dyson, Kathryn E Holt

**Affiliations:** 1 London School of Hygiene & Tropical Medicine, London, United Kingdom; 2 Department of Infectious Diseases, Central Clinical School, Monash University, Melbourne, Victoria, Australia; 3 Cambridge Institute of Therapeutic Immunology & Infectious Disease (CITIID), Department of Medicine, University of Cambridge, Cambridge, United Kingdom; 4 Wellcome Sanger Institute, Wellcome Genome Campus, Hinxton, Cambridge, United Kingdom

**Keywords:** AMR, pathogen genotyping, *Salmonella* Typhi, typhoid fever, WGS

## Abstract

In 2016, a whole-genome sequence (WGS)-based genotyping framework (GenoTyphi) was developed and provided a phylogenetically informative nomenclature for lineages of *Salmonella* Typhi, the etiological agent of typhoid fever. Subsequent surveillance studies have revealed additional epidemiologically important subpopulations, which require the definition of new genotypes and extension of associated software to facilitate the detection of antimicrobial resistance (AMR) mutations. Analysis of 4632 WGS provide an updated overview of the global *S* Typhi population structure and genotyping framework, revealing the widespread nature of haplotype 58 ([H58] 4.3.1) genotypes and the diverse range of genotypes carrying AMR mutations.

Typhoid fever is a fecal-orally transmitted systemic infection caused by the bacterium *Salmonella* Typhi. Each year >10 million cases occur worldwide, >100 000 of which are associated with mortalities [1], making it a public health threat in many low- to middle-income countries with limited hygiene and sanitation infrastructure.

*Salmonella* Typhi is a genetically monomorphic pathogen with a slow mutation rate that infrequently recombines [2]. Whole-genome sequencing (WGS) and core-genome phylogenetics have become the standard for typhoid molecular epidemiology in both research and public health settings, providing insights into population structure, transmission dynamics, antimicrobial resistance (AMR) emergence and dissemination, as well as outbreak investigation and monitoring of implemented intervention strategies. In 2016, a WGS-based genotyping framework for *S* Typhi was developed using a collection of ~2000 genomes from >60 countries [3], with the goal of stratifying the pathogen population and providing a phylogenetically informative nomenclature with which to refer to different lineages. The resulting scheme (known as GenoTyphi) utilized 68 marker single-nucleotide variants (SNVs) to define, based on an inferred genome-wide phylogeny, 4 primary clades, 16 clades, and 49 subclades organized in a pseudo-hierarchical nomenclature whereby primary clade 1 is subdivided into clades 1.1 and 1.2; clade 1.1 is further subdivided into subclades 1.1.1, 1.1.2, and 1.1.3. Haplotype 58 (H58), which has previously been associated with AMR and global dissemination via intercontinental transmission events [2], was designated genotype 4.3.1 under the new scheme. A software tool for calling GenoTyphi genotypes from WGS data was implemented in Python (available at https://github.com/katholt/genotyphi), facilitating integration of the scheme into bioinformatics pipelines. GenoTyphi is also available to nonexpert users via the online data analysis platform Typhi Pathogenwatch (https://pathogen.watch/) [4].

After publication of the initial framework, regional surveillance studies identified additional epidemiologically important subpopulations of *S* Typhi, which necessitated the definition of new genotypes [5–9]. Furthermore, point mutations responsible for reduced susceptibility to fluoroquinolones and azithromycin have also emerged [10, 11], necessitating extension of the GenoTyphi software tool for their detection. In this study, we provide an overview of updates to both the GenoTyphi scheme and pipeline (summarized in Supplementary Tables S1 and S2) as well as the view it provides of the global pathogen population.

## MATERIALS AND METHODS

### Phylogenetic and Single-Nucleotide Variant Analysis of *Salmonella* Typhi Isolates

Reads from 4632 *S* Typhi genomes (details in Supplementary Table S3 and Supplementary Methods) were mapped to the reference sequence of *S* Typhi CT18 (GenBank accession number AL513382) with RedDog (vbeta0.11; available at https://github.com/katholt/RedDog). Sequences were assigned to genotypes, and quinolone resistance-determining region (QRDR) and *acrB* mutations associated with AMR were detected, using GenoTyphi (v1.9.1; available at https://github.com/katholt/genotyphi), which is permanently archived by Zenodo [12]. Recombinant regions were removed from the whole-genome SNV alignment using Gubbins (v2.4.1; available at https://github.com/sanger-pathogens/gubbins) and a maximum-likelihood phylogeny inferred with RAxML (v8.2.9; available at https://github.com/stamatak/standard-RAxML). An interactive annotated phylogeny is available at https://microreact.org/project/vBoskUuenEVmfVzrcAMx8R. Further details are provided in Supplementary Methods.

## RESULTS

### Global Overview of *Salmonella* Typhi Genotypes

**Figure 1.**
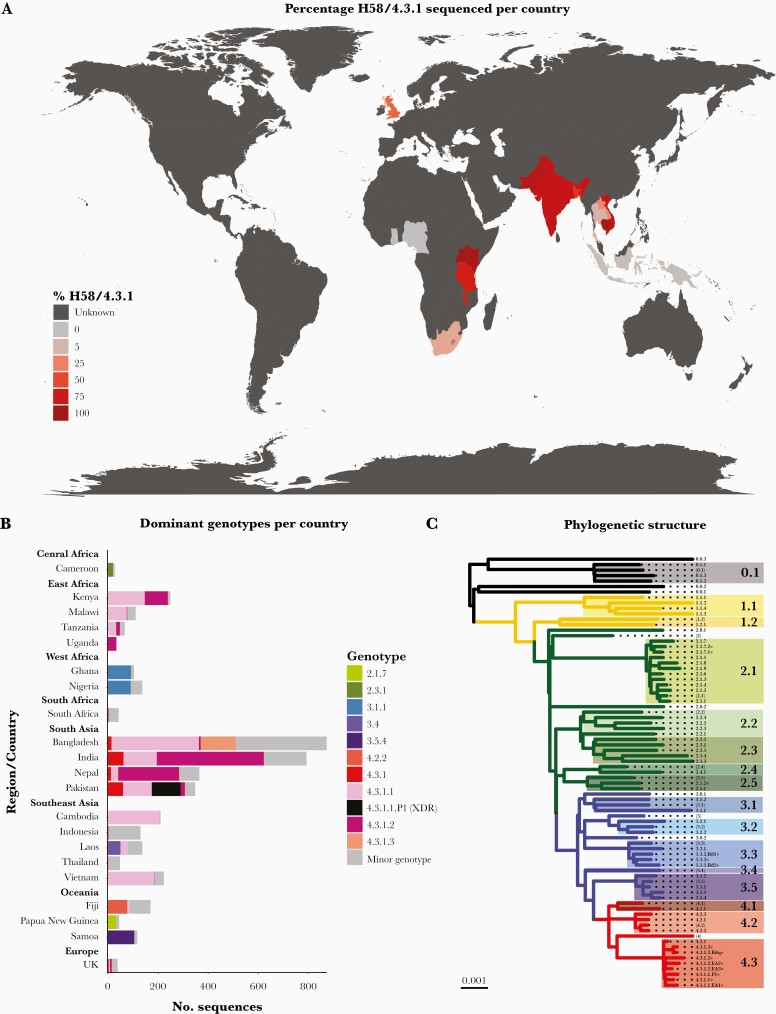
Global genotype distribution and population structure. (A) Global dissemination of genotype 4.3.1 (haplotype 58 [H58]). Countries are colored by total percentage of H58 genotypes among isolates in the genome collection where n ≥ 20 sequences were available, as per inset legend. Unknown indicates countries where 0–19 sequences were available. (B) Dominant genotypes per location. Dominant genotypes (each accounting for ≥30% of sequenced isolates per country) and H58 genotypes are colored as per the inset legend, with minor non-H58 genotypes in gray. Genotypes are shown for countries with at least 20 genome sequences. (C) Phylogenetic tree backbone showing the relationships between 16 clades and 63 subclades/sublineages. Tree tips represent unique genotypes as labeled, and background shading highlights clades (labeled in larger font). * indicates genotypes added to the scheme after its initial publication, and brackets indicate undifferentiated clades and primary clades. XDR, extensively drug resistant.

Analysis of 4632 published genomes demonstrate that H58 has now disseminated across most continents (Figure 1A), with the distribution of genotypes differing per country (Figure 1B). The 82 genotypes defined at present (Figure 1C and Supplementary Table S1) include those from the original publication, subdivision of 4.3.1 (H58) into 3 major lineages (4.3.1.1, 4.3.1.2, and 4.3.1.3), genotypes designating newly identified subclades (eg, 2.5.2 and 3.3.2), and designations for AMR populations of epidemiological importance (eg, 4.3.1.1.P1).

### Updated H58 (4.3.1) Genotypes

Genotype 4.3.1 is currently subdivided into 3 lineages (see Figure 1C and Supplementary Table S1). H58 lineages I (genotype 4.3.1.1) and II (genotype 4.3.1.2) were originally defined in a study of pediatric patients attending Patan Hospital in Kathmandu, Nepal [13]. Later studies [8] revealed the cocirculation of both lineages in this setting between 2008 and 2016, with a shift in dominance to 4.3.1.2 after 2010 (40% 4.3.1.2 pre-2010 and 74% post-2011, *P* = 1.0 × 10^7^), warranting more discriminant typing to capture such changes in population structure. H58 lineage III (genotype 4.3.1.3), originally defined in an examination of 536 AMR sequences from Dhaka, Bangladesh [6], is a monophyletic cluster of genotype 4.3.1 mostly from Bangladesh (99%). It was recently detected at a frequency of 9% in urban Dhaka between 2004 and 2016 [5]. A monophyletic sublineage of genotype 4.3.1.3 was resistant to fluoroquinolones (median minimal inhibitory concentration [MIC] of 4 μg/mL) and is here formally designated 4.3.1.3.Bdq based on previous studies [6].

A recent study of asymptomatic carriers and acute typhoid fever patients in Kenya detected the cocirculation of genotypes 4.3.1.1 and 4.3.1.2 [9]. Contextualization with the global phylogeny attributed the presence of these lineages to (1) two previously reported transmission waves originating in South Asia [2, 14] and (2) one third more recent introduction of 4.3.1.2 from South Asia that has apparently also reached Uganda [9]. These 3 H58 sublineages exclusively comprised East African sequences, had different AMR profiles, and were the result of separate introduction events, and thus they were designated as new genotypes to help monitor their spread: H58 lineage I sublineage East Africa I (4.3.1.1.EA1), H58 lineage II sublineage East Africa II (4.3.1.2.EA2), and H58 lineage II sublineage East Africa III (4.3.1.2.EA3) (Figure 1C and Supplementary Table S1) [9].

In 2016, outbreaks of the first widespread extensively drug resistant clone occurred in Pakistan. This monophyletic outbreak cluster of genotype 4.3.1.1—resistant to chloramphenicol, ampicillin, co-trimoxazole, fluroquinolones, and third-generation cephalosporins [7, 15]—was designated genotype 4.3.1.1.P1 to aid its identification.

### Updated Non-H58 Genotypes

Studies of *S* Typhi in Bangladesh [5] revealed that 119 genomes (14.5% of sequences analyzed) formed a monophyletic group of sequences typed only to the clade level (genotype 3.3) that was related to sequences from Nepal (separated by ~70 SNVs, also typed as 3.3). These were collectively designated genotype 3.3.2. Within the Bangladesh 3.3.2, two sublineages carrying QRDR mutations were further defined to facilitate their detection in future surveillance studies: 3.3.2.Bd1 (which typically carry *gyrA*-S83F) and 3.3.2.Bd2 (which typically carry *gyrA*-S87N) (see Figure 1C and Supplementary Table S1).

Ongoing analysis of genomes from Madagascar and Papua New Guinea ([PNG] to be described in detail elsewhere) have also identified localized variants. The Madagascar group belongs to clade 2.5, is distantly related to other 2.5 sequences from India (separated by ~122 SNVs), and has been designated 2.5.2. The PNG genotype 2.1.7 population is subdivided into 2 distinct sublineages designated genotypes, 2.1.7.1 and 2.1.7.2, with 2.1.7.1 observed more frequently.

### Updated Detection of Resistance-Associated Mutations

Aforementioned studies of pediatric typhoid in Kathmandu, Nepal revealed high levels (75.3%) of sequences carrying nonsynonymous point mutations in the QRDR of genes *gyrA*, *gyrB*, and *parC* responsible for reduced susceptibility to fluoroquinolones from 2008 to 2016 [8]. Among these were sequences of genotype 4.3.1.2 carrying 3 such mutations (eg, *gyrA*-S83F, *gyrA*-D87N, *parC*-S80I – 7.6%; *gyrA*-S83F, *gyrA*-D87N, *parC*-E84K – .5%), the former of which was previously found to cause treatment failure among adult populations in the same setting [11]. More recent studies [10] demonstrated that mutations at codon 717 of gene *acrB*, a component of the AcrAB-TolC drug efflux pump, mediate azithromycin resistance (MIC ≥32 μg/mL) in *S* Typhi and had been observed at low frequency in Dhaka, Bangladesh (~1.3% of all *S* Typhi isolated from 2009 to 2016). Subsequently, the GenoTyphi pipeline has been extended to detect mutations in both the QRDR and codon 717 of gene *acrB* (see Supplementary Table S2).

### Global Overview of Antimicrobial Resistance-Associated Mutations

**Figure 2.**
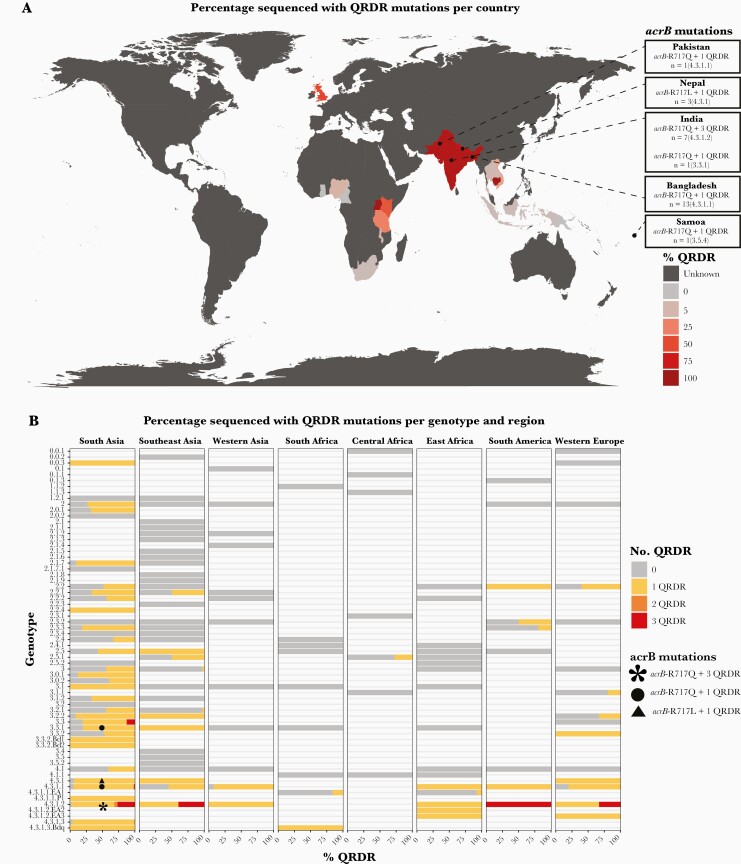
Global overview of antimicrobial resistance mutations. (A) Global distribution of quinolone resistance-determining region (QRDR) mutations. Countries are colored by the total percentage of sequences carrying QRDR mutations in the genome collection where n ≥ 20 sequences were available, as per inset legend. Unknown indicates countries where 0–19 sequences were available. Locations where sequences also carrying *acrB*-R717Q/L mutations have been isolated are indicated as labeled. (B) Distribution of QRDR mutations by genotype and region. Genotype/region combinations are included where ≥20 isolates have been sequenced from the region and ≥5% of those carry QRDR mutations. Genotypes also carrying *acrB*-R717Q/L mutations are labeled as per the inset legend.

Analysis of published genomes demonstrates that sequences carrying QRDR mutations can now be found across most continents (Figure 2A), with the diversity of genotypes carrying QRDR mutations varying by geographic region (Figure 2B). The geographic distribution of sequences carrying *acrB*-R717Q/L mutations associated with azithromycin resistance are shown in Figure 2A and B. These mutations have emerged independently in multiple *S* Typhi genotypes in several different countries, mostly in South Asia at present, and are accompanied by QRDR mutations, making them coresistant to fluoroquinolones (see Figure 2B). Isolates from Dhaka [10] have also been reported to be multidrug resistant, carrying genes that confer additional resistance to chloramphenicol, ampicillin, and co-trimoxazole. Recent studies [16] have revealed that these mutations now appear to be emerging in more non-H58 genotypes in Dhaka from 2016 onwards including genotypes 2.3.3, 3.2.2, and 3.3.2.

## DISCUSSION

In the 5 years since the publication of the GenoTyphi framework, several regional genomic surveillance studies have been carried out, providing further insight into transmission events on a regional and global scale (including the continued global spread of 4.3.1 genotypes, and the emergence, spread, and ongoing evolution of mutations responsible for AMR in a diverse range of H58 and non-H58 genotypes) and the identification of new genotypes (Supplementary Tables S1 and S2). The published datasets aggregated here span multiple studies carried out over different time periods utilizing different sampling strategies, and, as such, the data presented here cannot be considered representative of national genotype and AMR frequencies. The use of public data for such estimates would be facilitated by adoption of metadata standards that convey whether genome collections represent unbiased surveillance, as proposed by the newly formed Global Typhoid Genomics Consortium (https://www.typhoidgenomics.org/).

## CONCLUSIONS

The GenoTyphi framework will continue to be developed as new data become available and as new variants emerge, providing up-to-date phylogenetically informative nomenclature for identifying and discussing trends in population structure and evolution of AMR in *S* Typhi. This nomenclature remains critical in genetic epidemiology studies required for the successful implementation and monitoring of control strategies. To date, new genotypes have been included on the basis of publications identifying subpopulations as having epidemiological importance [5–9, 15]. However, requests for the inclusion of new genotypes can also be made via the GitHub repository (https://github.com/katholt/genotyphi), and in future more systematic criteria and processes for inclusion of new genotypes will be overseen by the Global Typhoid Genomics Consortium steering committee.

## Supplementary Data

Supplementary materials are available at *The Journal of Infectious Diseases* online. Supplementary materials consist of data provided by the author that are published to benefit the reader. The posted materials are not copyedited. The contents of all supplementary data are the sole responsibility of the authors. Questions or messages regarding errors should be addressed to the author.

**Supplementary Table S1.** Summary of *S* Typhi genotypes (Excel spreadsheet). “Reference allele” indicates the allele in the CT18 reference sequence. “Alternative allele” indicates an allele called against the CT18 reference sequence for the genotype called. “Derived allele” indicates the subtree-defining allele, which resulted from mutation of the original (ancestral) allele at this position to generate a new (derived) allele that we use as the marker for the subtree that corresponds to this genotype. “Ancestral allele” indicates the allele present in the ancestor of S Typhi, which is conserved by all members of the population outside of the subtree that corresponds to this genotype.

**Supplementary Table S2.** Summary of *S* Typhi AMR mutations detected by GenoTyphi (Excel spreadsheet). “Reference allele” indicates the allele in the CT18 reference sequence. “Alternative allele” indicates an allele called against the CT18 reference sequence for the genotype called.

**Supplementary Table S3.** Details of publicly available *S* Typhi genome sequences analyzed in this study (Excel spreadsheet).

jiab414_suppl_Supplementary_Materials

jiab414_suppl_Supplementary_Table_S1

jiab414_suppl_Supplementary_Table_S2

jiab414_suppl_Supplementary_Table_S3

## Notes

***Financial support.*** Z. A. D. was supported by a grant funded by the Wellcome Trust (STRATAA; Grant Number 106158/Z/14/Z) and received funding from the European Union’s Horizon 2020 research and innovation programme under the Marie Skłodowska-Curie grant agreement TyphiNET (Grant Number 845681). K. E. H. was supported by a Senior Medical Research Fellowship from the Viertel Foundation of Australia and the Bill & Melinda Gates Foundation (Grant Number OPP1175797).

***Potential conflicts of interest.*** All authors: No reported conflicts of interest. All authors have submitted the ICMJE Form for Disclosure of Potential Conflicts of Interest.

Presented in part: 15th Asian Conference on Diarrhoeal Disease and Nutrition (ASCODD), Dhaka, Bangladesh, January 30, 2020.
