# Supplementary material for: Five Years of GenoTyphi: Updates to the Global Salmonella Typhi Genotyping Framework
Source: J Infect Dis. 2021 Aug 28;224(Suppl 7):S775–80. doi: 10.1093/infdis/jiab414 (PMC8687072; doi:10.1093/infdis/jiab414)
Supplement: jiab414_suppl_Supplementary_Materials [file jiab414_suppl_supplementary_materials.docx]

**Supplementary methods**

***Phylogenetic and SNV analysis of S. Typhi isolates***

An extensive literature search was carried out to identify published *S.* Typhi whole genome sequences (WGS) with both metadata (at least country and year of isolation) and raw read data made publicly available prior to February 2021. This yielded a total of 4,632 *S.* Typhi genomes (detailed in **Table S3**) ^1-20^ which were subsequently obtained from the European Nucleotide Archive (ENA). For Single Nucleotide Variant (SNV) analysis, paired-end reads were mapped to the reference sequence of *S.* Typhi CT18 (accession number: AL513382)^21^ using the RedDog mapping pipeline (vbeta.11), available at: <https://github.com/katholt/RedDog>. RedDog uses Bowtie (v2.2.3)^22^ to map reads to the reference sequence; SAMtools (v0.1.19)^23^ to identify SNVs with phred quality scores above 30; to filter out SNVs supported by <5 reads, or with 2.5 times the genome-wide average read depth (representing putative repeated sequences), or with ambiguous (heterozygous) consensus base calls. For every SNV position that passes the aforementioned criteria in any one isolate, consensus base calls (i.e. alleles) for that position were extracted from all genomes analysed, and used to construct an alignment of alleles across all SNV sites. Resultant read alignments (BAM format) were then used to assign sequences to previously defined genotypes (**Table S1**) according to the *S.* Typhi extended genotyping framework with the GenoTyphi (v1.9.1) pipeline (available at: <https://github.com/katholt/genotyphi>)^18^ which is permanently archived by Zenodo^24^ at doi: 10.5281/zenodo.4707614. AMR mutations in the quinolone resistance determining region (QRDR) and at codon 717 of gene *acrB* (detailed in **Table S2**) were detected in the same manner with GenoTyphi.

For phylogenetic analyses, chromosomal SNVs with confident homozygous calls (phred score >20) in more than 95% of the genomes mapped (representing a “soft” core genome) were concatenated to form an alignment of alleles at 36,859 variant sites, and alleles

from *S.* Paratyphi A str. AKU_12601^25^ (accession number: FM200053) were also included in the phylogenetic analysis as an outgroup for tree rooting. SNVs called in phage regions or repetitive sequences (354 kbp; ~7.4% of bases in the CT18 reference sequence, defined previously^5^,^18^,^26^) were filtered from the alignment, which was then used with the CT18 reference genome to produce a whole genome pseudoalignment that was subjected to recombination filtering with Gubbins (v2.4.1)^27^ to remove any further recombinant regions. This resulted in a final set of 35,599 SNVs identified in a final alignment length of 4,275,037 sites for the 4,633 sequences.

From the global SNV alignment, a maximum likelihood (ML) phylogenetic tree was inferred using RAxML (v8.2.9)^28^, with a generalised time-reversal model, a Gamma distribution to model site-specific rate variation (the GTR+ Γ substitution model; GTRGAMMA in RAxML), and 100 bootstrap pseudo-replicates to assess branch support.

Phylogenetic trees were visualised using Microreact^29^ and the R package ggtree (v2.2.4)^30^. An interactive phylogeny annotated with AMR mutations and genotypes is available at: <https://microreact.org/project/vBoskUuenEVmfVzrcAMx8R>. For the purpose of plotting the global genotype structure in **Fig. 1c**, clusters of *S.* Typhi in the full tree that were members of the same genotype were collapsed into a single representative each using the *drop.tip()* function in the R package ape^31^.

**References**

1 Britto Carl D, Dyson Zoe A, Duchene Sebastian, Carter Michael J, Gurung Meeru, Kelly Dominic F, et al. Laboratory and molecular surveillance of paediatric typhoidal Salmonella in Nepal: Antimicrobial resistance and implications for vaccine policy. *PLoS Negl Trop Dis* 2018;**12**(4):e0006408. Doi: 10.1371/journal.pntd.0006408.

2 Britto Carl D, Dyson Zoe A, Mathias Sitarah, Bosco Ashish, Dougan Gordon, Jose Sanju, et al. Persistent circulation of a fluoroquinolone-resistant Salmonella enterica Typhi clone in the Indian subcontinent. *J Antimicrob Chemother* 2020;**75**(2):337–41. Doi: 10.1093/jac/dkz435.

3 Carey Megan E, Jain Ruby, Yousuf Mohammad, Maes Mailis, Dyson Zoe A, Thu Trang Nguyen Hoang, et al. Spontaneous Emergence of Azithromycin Resistance in Independent Lineages of Salmonella Typhi in Northern India. *Clin Infect Dis* 2021;**72**(5):e120–7. Doi: 10.1093/cid/ciaa1773.

4 Hooda Yogesh, Sajib Mohammad S I, Rahman Hafizur, Luby Stephen P, Bondy-Denomy Joseph, Santosham Mathuram, et al. Molecular mechanism of azithromycin resistance among typhoidal Salmonella strains in Bangladesh identified through passive pediatric surveillance. *PLoS Negl Trop Dis* 2019;**13**(11):e0007868. Doi: 10.1371/journal.pntd.0007868.

5 Ingle Danielle J, Nair Satheesh, Hartman Hassan, Ashton Philip M, Dyson Zoe A, Day Martin, et al. Informal genomic surveillance of regional distribution of Salmonella Typhi genotypes and antimicrobial resistance via returning travellers. *PLoS Negl Trop Dis* 2019;**13**(9):e0007620. Doi: 10.1371/journal.pntd.0007620.

6 International Typhoid Consortium, Wong Vanessa K, Holt Kathryn E, Okoro Chinyere, Baker Stephen, Pickard Derek J, et al. Molecular Surveillance Identifies Multiple Transmissions of Typhoid in West Africa. *PLoS Negl Trop Dis* 2016;**10**(9):e0004781. Doi: 10.1371/journal.pntd.0004781.

7 Iqbal Junaid, Dehraj Irum F, Carey Megan E, Dyson Zoe A, Garrett Denise, Seidman Jessica C, et al. A Race against Time: Reduced Azithromycin Susceptibility in Salmonella enterica Serovar Typhi in Pakistan. *mSphere* 2020;**5**(4). Doi: 10.1128/mSphere.00215-20.

8 Klemm Elizabeth J, Shakoor Sadia, Page Andrew J, Qamar Farah Naz, Judge Kim, Saeed Dania K, et al. Emergence of an Extensively Drug-Resistant Salmonella entericaSerovar Typhi Clone Harboring a Promiscuous Plasmid Encoding Resistance to Fluoroquinolones and Third-Generation Cephalosporins. *mBio* 2018;**9**(1):346–17. Doi: 10.1128/mBio.00105-18.

9 Maes Mailis, Dyson Zoe A, Higginson Ellen E, Fernandez Alda, Araya Pamela, Tennant Sharon M, et al. Multiple Introductions of SalmonellaentericaSerovar Typhi H58 with Reduced Fluoroquinolone Susceptibility into Chile. *Emerg Infect Dis* 2020;**26**(11):2736–40. Doi: 10.3201/eid2611.201676.

10 Matono Takashi, Morita Masatomo, Yahara Koji, Lee Ken-ichi, Izumiya Hidemasa, Kaku Mitsuo, et al. Emergence of Resistance Mutations in Salmonella enterica Serovar Typhi Against Fluoroquinolones. *Open Forum Infect Dis* 2017;**4**(4):ofx230. Doi: 10.1093/ofid/ofx230.

11 Park Se Eun, Pham Duy Thanh, Boinett Christine, Wong Vanessa K, Pak Gi Deok, Panzner Ursula, et al. The phylogeography and incidence of multi-drug resistant typhoid fever in sub-Saharan Africa. *Nat Commun* 2018;**9**(1):5094. Doi: 10.1038/s41467-018-07370-z.

12 Pragasam Agila Kumari, Pickard Derek, Wong Vanessa, Dougan Gordon, Kang Gagandeep, Thompson Andrew, et al. Phylogenetic Analysis Indicates a Longer Term Presence of the Globally Distributed H58 Haplotype of Salmonella Typhi in Southern India. *Clin Infect Dis* 2020;**71**(8):1856–63. Doi: 10.1093/cid/ciz1112.

13 Rahman Sadia Isfat Ara, Dyson Zoe A, Klemm Elizabeth J, Khanam Farhana, Holt Kathryn E, Chowdhury Emran Kabir, et al. Population structure and antimicrobial resistance patterns of Salmonella Typhi isolates in urban Dhaka, Bangladesh from 2004 to 2016. *PLoS Negl Trop Dis* 2020;**14**(2):e0008036. Doi: 10.1371/journal.pntd.0008036.

14 Rasheed Farhan, Saeed Muhammad, Alikhan Nabil-Fareed, Baker David, Khurshid Mohsin, Ainsworth Emma V, et al. Emergence of Resistance to Fluoroquinolones and Third-Generation Cephalosporins in Salmonella Typhi in Lahore, Pakistan. *Microorganisms* 2020;**8**(9). Doi: 10.3390/microorganisms8091336.

15 Tanmoy Arif M, Westeel Emilie, De Bruyne Katrien, Goris Johan, Rajoharison Alain, Sajib Mohammad S I, et al. Salmonella enterica Serovar Typhi in Bangladesh: Exploration of Genomic Diversity and Antimicrobial Resistance. *mBio* 2018;**9**(6). Doi: 10.1128/mBio.02112-18.

16 Pham Thanh Duy, Karkey Abhilasha, Dongol Sabina, Ho Thi Nhan, Thompson Corinne N, Rabaa Maia A, et al. A novel ciprofloxacin-resistant subclade of H58 Salmonella Typhi is associated with fluoroquinolone treatment failure. *Elife* 2016;**5**:e14003. Doi: 10.7554/eLife.14003.

17 Duy P T, Dongol S, Giri A, Nguyen Thi Nguyen To, Ngoc Dan Thanh Ho, Nguyen Quynh Nhu Pham, et al. The emergence of azithromycin-resistant Salmonella Typhi in Nepal. *JAC-Antimicrobial Resistance* 2020;**2**(4). Doi: 10.1101/2020.08.07.20166389.

18 Wong Vanessa K, Baker Stephen, Connor Thomas R, Pickard Derek, Page Andrew J, Dave Jayshree, et al. An extended genotyping framework for Salmonella enterica serovar Typhi, the cause of human typhoid. *Nat Commun* 2016;**7**:12827. Doi: 10.1038/ncomms12827.

19 Kariuki Samuel, Dyson Zoe A, Mbae Cecilia, Ngetich Ronald, Kavai Susan M, Wairimu Celestine, et al. Multiple introductions of multidrug-resistant typhoid associated with acute infection and asymptomatic carriage, Kenya. *bioRxiv* 2021:2021.03.10.434750. Doi: 10.1101/2021.03.10.434750.

20 Dyson Zoe A, Thanh Duy Pham, Bodhidatta Ladaporn, Mason Carl Jeffries, Srijan Apichai, Rabaa Maia A, et al. Whole Genome Sequence Analysis of Salmonella Typhi Isolated in Thailand before and after the Introduction of a National Immunization Program. *PLoS Negl Trop Dis* 2017;**11**(1):e0005274. Doi: 10.1371/journal.pntd.0005274.

21 Parkhill J, Dougan G, James K D, Thomson N R, Pickard D, Wain J, et al. Complete genome sequence of a multiple drug resistant Salmonella enterica serovar Typhi CT18. *Nature* 2001;**413**(6858):848–52. Doi: 10.1038/35101607.

22 Langmead Ben, Salzberg Steven L. Fast gapped-read alignment with Bowtie 2. *Nat Meth* 2012;**9**(4):357–9. Doi: 10.1038/nmeth.1923.

23 Li Heng, Handsaker Bob, Wysoker Alec, Fennell Tim, Ruan Jue, Homer Nils, et al. The Sequence Alignment/Map format and SAMtools. *Bioinformatics* 2009;**25**(16):2078–9. Doi: 10.1093/bioinformatics/btp352.

24 Dyson Zoe Anne, Holt Kathyrn E. katholt/genotyphi: GenoTyphi v1.9.1 2021. Doi: 10.5281/zenodo.4707614.

25 Holt Kathryn E, Thomson Nicholas R, Wain John, Langridge Gemma C, Hasan Rumina, Bhutta Zulfiqar A, et al. Pseudogene accumulation in the evolutionary histories of Salmonella enterica serovars Paratyphi A and Typhi. *BMC Genomics* 2009;**10**:36. Doi: 10.1186/1471-2164-10-36.

26 Holt Kathryn E, Parkhill Julian, Mazzoni Camila J, Roumagnac Philippe, Weill François-Xavier, Goodhead Ian, et al. High-throughput sequencing provides insights into genome variation and evolution in Salmonella Typhi. *Nat Genet* 2008;**40**(8):987–93. Doi: 10.1038/ng.195.

27 Croucher Nicholas J, Page Andrew J, Connor Thomas R, Delaney Aidan J, Keane Jacqueline A, Bentley Stephen D, et al. Rapid phylogenetic analysis of large samples of recombinant bacterial whole genome sequences using Gubbins. *Nucleic Acids Research* 2015;**43**(3):e15–5. Doi: 10.1093/nar/gku1196.

28 Stamatakis Alexandros. RAxML version 8: a tool for phylogenetic analysis and post-analysis of large phylogenies. *Bioinformatics* 2014;**30**(9):1312–3. Doi: 10.1093/bioinformatics/btu033.

29 Argimon Silvia, Abudahab Khalil, Goater Richard J E, Fedosejev Artemij, Bhai Jyothish, Glasner Corinna, et al. Microreact: visualizing and sharing data for genomic epidemiology and phylogeography. *Microbial Genomics* 2016;**2**(11):e000093. Doi: 10.1099/mgen.0.000093.

30 Yu Guangchuang, Smith David K, Zhu Huachen, Guan Yi, Lam Tommy Tsan-Yuk. ggtree: an rpackage for visualization and annotation of phylogenetic trees with their covariates and other associated data. *Methods Ecol Evol* 2016;**8**(1):28–36. Doi: 10.1111/2041-210X.12628.

31 Paradis Emmanuel, Claude Julien, Strimmer Korbinian. APE: Analyses of Phylogenetics and Evolution in R language. *Bioinformatics* 2004;**20**(2):289–90. Doi: 10.1093/bioinformatics/btg412.
